# Three minimum tile paths from bacterial artificial chromosome libraries of the soybean (*Glycine max *cv. 'Forrest'): tools for structural and functional genomics

**DOI:** 10.1186/1746-4811-2-9

**Published:** 2006-05-25

**Authors:** JL Shultz, C Yesudas, S Yaegashi, AJ Afzal, S Kazi, DA Lightfoot

**Affiliations:** 1 Dept of Soybean Genetics, United States Department of Agriculture, Stoneville, MS 38776, USA; 2 Dept. of Plant Soil and Agricultural Systems, Genomics and Biotechnology Facility, Center for Excellence in Soybean Research, Southern Illinois University, Carbondale, IL 62901, USA; 3 Dept of Bioinformatics, University of Tokyo, Tokyo, Japan

## Abstract

**Background:**

The creation of minimally redundant tile paths (hereafter MTP) from contiguous sets of overlapping clones (hereafter contigs) in physical maps is a critical step for structural and functional genomics. Build 4 of the physical map of soybean (*Glycine max *L. Merr. cv. 'Forrest') showed the 1 Gbp haploid genome was composed of 0.7 Gbp diploid, 0.1 Gbp tetraploid and 0.2 Gbp octoploid regions. Therefore, the size of the unique genome was about 0.8 Gbp. The aim here was to create MTP sub-libraries from the soybean cv. Forrest physical map builds 2 to 4.

**Results:**

The first MTP, named MTP2, was 14,208 clones (of mean insert size 140 kbp) picked from the 5,597 contigs of build 2. MTP2 was constructed from three BAC libraries (*Bam*HI (B), *Hin*dIII (H) and *Eco*RI (E) inserts). MTP2 encompassed the contigs of build 3 that derived from build 2 by a series of contig merges. MTP2 encompassed 2 Gbp compared to the soybean haploid genome of 1 Gbp and does not distinguish regions by ploidy. The second and third MTPs, called MTP4BH and MTP4E, were each based on build 4. Each was semi-automatically selected from 2,854 contigs. MTP4BH was 4,608 B and H insert clones of mean size 173 kbp in the large (27.6 kbp) T-DNA vector pCLD04541. MTP4BH was suitable for plant transformation and functional genomics. MTP4E was 4,608 BAC clones with large inserts (mean 175 kbp) in the small (7.5 kbp) pECBAC1 vector. MTP4E was suitable for DNA sequencing. MTP4BH and MTP4E clones each encompassed about 0.8 Gbp, the 0.7 Gbp diploid regions and 0.05 Gbp each from the tetraploid and octoploid regions. MTP2 and MTP4BH were used for BAC-end sequencing, EST integration, micro-satellite integration into the physical map and high information content fingerprinting. MTP4E will be used for genome sequence by pooled genomic clone index.

**Conclusion:**

Each MTP and associated BES will be useful to deconvolute and ultimately finish the whole genome shotgun sequence of soybean.

## Background

The construction of a fingerprint-based physical map in soybean (*Glycine max *L. Merr.) has relied on three large insert genomic libraries [1,2]. Two large insert DNA libraries were constructed in the pCLD04541, a 27.6 Kbp *oriT *based low copy per cell, T-DNA with *nptII*, binary, tetracycline resistance conferring plasmid vector [Genbank # AF184978; 3]. Partial digestion of high molecular weight genomic DNA was with *Bam*HI (B) or *Hin*dIII (H) [1]. One large insert DNA libraries was constructed in the pECBAC1 vector [4], a 7.5 kbp *oriS *based single copy per cell, chloramphenicol resistance conferring plasmid vector. Partial digestion of high molecular weight genomic DNA was with *Eco*RI (E) [2]. The mean insert sizes across libraries were 125 ± 5 kbp (B), 135 ± 5 kbp (H) and 157 ± 10 kbp (E). All three libraries contained a substantial proportion (10–20%) of BAC clones with inserts of 160–240 kbp. Larger clones than 240 kbp existed [3] but most were shown to be chimaeras and contaminants that had to be removed for physical map development [5].

The physical map of soybean was developed from sequencing PAGE separation of *Hin*dIII, *Hae*III restriction digest fragments [6]. About 35–50 bands per clone could be used for contig builds by FPC [7]. The use of three BAC libraries generated with three different restriction enzymes in two different plasmid vectors has avoided biases in genome representation [2]. The fingerprint method has several advantages over agarose gel and capillary sequencing gel fingerprinting [8].

Build 1, the first publicly available physical map tool for soybean, was based on 30,000 BAC fingerprints. The data were available for a short time [9]. Build 2 appeared to resolve clone identification issues from Build 1, and consisted of 5,597 contigs (Table 1). The contigs were built at varying cut-off stringencies (e^-18^-e^-28^). Build 3 contigs derived from build 2 contigs by manual editing that included merging potentially overlapping contigs and splitting contigs with more than 12 BACs per unique band [2,6,9,10]. In addition to 2,901 contigs (Table 1), build three incorporated DNA markers and was the first functional build available to public soybean research. Build 3 contigs were provided for viewing through a genome browser interface at SoyGD [10] in the context of soybean genome information including DNA markers, BAC end sequence, QTL and EST hybridizations [11,12].

**Table 1:** Progress in the soybean physical map (builds 2 to 4).

	Build 2	Build 3	Build 4	Build 4 repeats	Footnotes
BAC clones in FPC database	81,024	78,001	78,001		
BACs used in contig assembly	75,568	76,749	72,942		1, 2
Number of singletons	5,884	3,702	27,810		
Marker anchored singletons	0	0	120		
Clones in contigs (fold genome)	69,684	73,047	45,130		
Fold genome in contigs	8.7	9.1	5.6		
Number of contigs	5,597	2,905	2,854	646	
Anchoring Markers	0	385	404		
Anchored Contigs	0	781	742	181	
Q Contigs	n/a	1040	0		
Contigs contain:					
> 25 clones	220	921	477	268	3
10 – 25 clones	3,038	920	1,458	433	4
3 – 9 clones	1,845	850	820	0	
2 clones	385	216	99	0	
Unique bands within contigs	396,843	345,457	258,240	54,560	5
Length of the contigs (Mb)	16,676	14,516	1.037	0.258	

^1 ^Adjusted for 1252 "NoFp" 0 band clones only (includes all clones with > = 1 band).

^2 ^Adjusted for all 0–4 band clones, contamination-suspect clones and most high band number (>65) clones.

^3 ^All repeat contigs with greater than 25 clones are represented in contig #'s 9000 and greater.

^4 ^Includes 265 repeat contigs that have 15–19 clones (contig #'s 8000 – 8264) and 168 contigs that have 20–24 clones in contig #'s 9000 and greater.

^5 ^Based on 4.0 kbp per unique band, for 2,208 contigs containing ~68 unique bands in 15 clones, 264 duplicated region contigs containing ~68 unique bands in 30 clones 15,840 unique bands and 406 highly repeated region contigs containing ~68 unique bands in 60 clones, 48,720 unique bands.

^6 ^Based on 4.0 kbp per unique band.

There were three reasons to create an improved build of the soybean genome (build 4); detection of contig merge and split errors during manual re-examination of the soybean libraries for MTP selection [5]; the detection of potential neighboring well contaminants that cause false merges [5]; and the concern that the build 3 map encompassed 1.4 Gbp, about 0.3 Gbp more than the soybean genome [2,6,9].

The result of Build 4 was 2,854 contigs (Table 1). Build 4 contigs were provided for viewing through a separate window to build 3 in the SoyGD genome browser interface [10]. Build 4 is shown in the context of soybean genome information including DNA markers, BAC end sequence, QTL and EST hybridizations [11,12]. Development of robust contig sets and increased demands for libraries and filter sets from the research community prompted the development of minimally redundant tile paths (MTP).

MTPs derived from physical maps have been used widely among genomes where physical maps exist [7,13]. MTPs may be used to develop whole genome sequences [14-17] and to examine synteny among genomes [18]. MTPs were used to identify chromosomal rearrangements [19] and to determine the timing of DNA replication among chromosomes [20]. MTPs are useful for genetic map development [21], gene map development [22,23] and positional cloning [12,24]. In each area of use the ability to use MTP BAC clones for cell transformation can be important.

Binary vectors have been used to develop BAC libraries for fungi, plants and animals [17,25,26]. The libraries facilitate *Agrobacterium*-mediated transformation with large DNA fragments to fungal [27], plant [25,28] and animal [29] cells. The stability of different clones varies with insert size, origin of replication, cloning site and *Agrobacterium *strain [2,6,30]. Therefore to realize the potential of clones for functional genomics, a heterogeneous series of clones covering an interval should be used. Overlapping sets of MTP derived from physical maps built from BAC fingerprints can provide a heterogeneous series of clones covering an interval [2,6].

The creation of soybean minimum tiling path libraries (MTPs) was undertaken in order to reduce the number of clones required for functional and structural genomics. Uses have included EST integration, high information content fingerprinting (HICF), high throughput marker integration, BAC-end sequence synteny analysis and soybean genome sequence.

## Results and discussion

**Figure 2:**
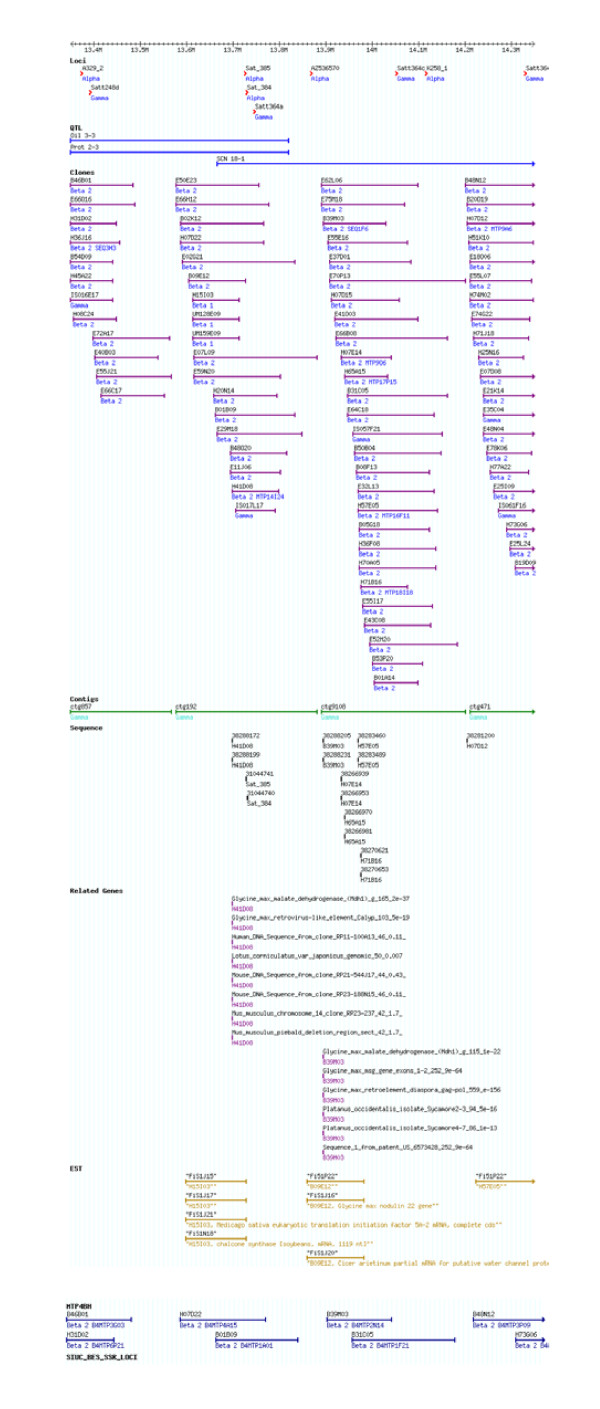
Gbrowse representation at SoyGD of the MTP clones in a portion of the soybean genome from linkage group A1 from 13.35 to 14.35 Mbp. A 1 Mbp region of build 4 with loci, QTL, clones, contigs, sequences and gene models are shown. Loci, or genetic map DNA markers, are shown as red arrow heads. QTL in the region are shown as blue bars. BAC clones are shown as purple bars. BAC clones are annotated above the bar with their master plate address and below the bar with the clone classification. Gamma clones were linked to a locus, betas were linked to a gamma clone, alphas were an unlinked feature. MTP4 clones in build 4 were annotated below the bar with the MTP plate address. MTP2 clones included in build 4 can be identified as they have BES and EST hits shown. Contigs are shown as green bars. Polyploid region contigs have ctg numbers greater than 8,000. Sequences from MTP BAC ends are shown as black lines. Related gene annotations are shown as purple lines (the 5 most probable Blastx hits at P < e-5 are listed). ESTs mapped to MTP BACs are shown as golden bars and annotated with master plate address and gene model (if known) below the bar and EST name above the bar. Clicking on EST or MTP clones brings up the gene index number.

Three MTP sub-libraries were created. All three MTP can be viewed in Gbrowse at SoyGD (Figure 2). Filters and plates containing the MTPs are available on request at significantly lower cost than whole libraries.

### MTP2

The MTP2 consisted of thirty seven 384 well plates (13 *Hind*III, 16 *Eco*RI and 8 *Bam*HI) created from 5,597 contigs (supplementary Table 2). The 14,208 clones of MTP2 appeared to have a mean insert size of 140 ± 5 kbp as judged by 35 useful bands per fingerprint, and by agarose gel electrophoresis after digestion with *Not*I, *Hind*III, *Eco*RI or *Bam*HI (not shown). The 140 Kbp insert size is intermediate between the insert sizes of the three contributing libraries and may be a conservative estimate. Therefore, MTP2 encompassed at least 2 Gbp compared to the soybean haploid genome of 1 Gbp. Each region of the genome should be represented twice. However, since build 2 and 3 did not distinguish regions by ploidy some highly conserved tetraploid and octoploid regions may be represented 4–8 fold.

MTP2 has been used since 2003 for BAC-end sequencing, EST hybridization, DNA micro-satellite marker integration into the physical map and positional cloning [11,12,21,23,31].

### MTP4BH

**Figure 3:**
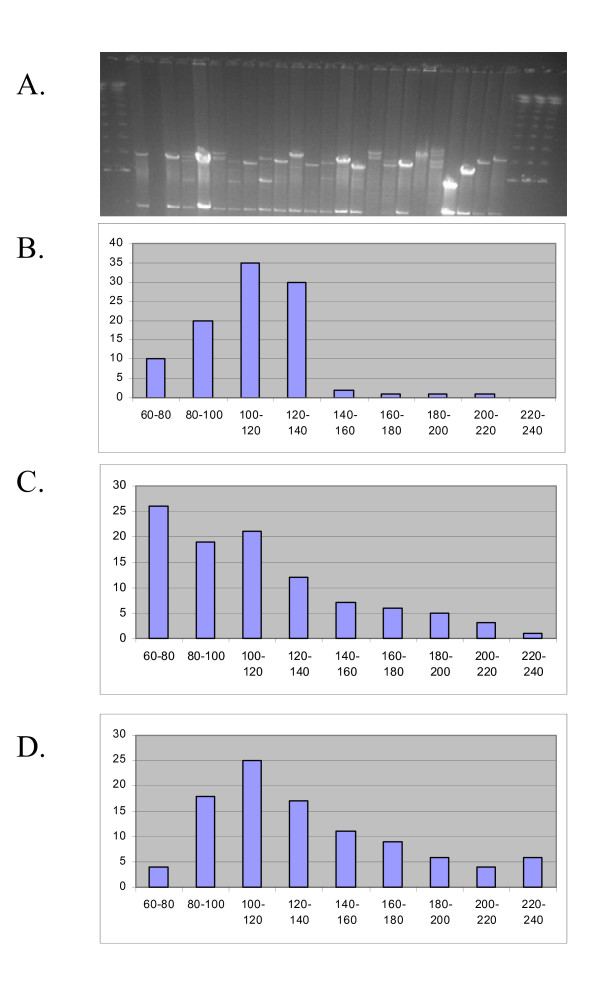
Comparison of BAC insert size in source libraries compared to the minimum tile paths. Panel A shows a pulsed field gel electrophoresis of 24 MTP4BH BACs from the *Bam*H1 library. Panel B shows the insert size range for the 100 BACs from B library reported in Meksem et al., 2000. Panel C shows the insert size range for the 100 BACs from the B library as measured from Panel A plus 4 additional gels. Panel D shows the insert size range for the 100 BACs from the MTP4BH library as measured from Panel A and additional gels of solely MTP clones after re-arraying (not shown).

The second and third MTPs, called MTP4BH and MTP4E, were each based on build 4. Each was semi-automatically selected from 2,854 contigs. MTP4BH was 12 plates that contained 4,608 *Hind*III or *Bam*HI insert clones. The inserts of selected clones were of mean size 173 kbp as judged by 43 useful bands per fingerprint, and by agarose gel electrophoresis after digestion with *Not*I, *Hind*III or *Bam*HI (Figure 3). The 173 Kbp insert size is much larger (38–48 Kbp) than the mean insert sizes of the 2 contributing libraries suggesting large insert BACs were selected and small insert BACs avoided. Therefore, MTP4BH encompassed about 0.8 Gbp compared to the soybean haploid genome of 1 Gbp. MTP4BH encompassed about 0.7 Gbp from the 4,032 clones selected from diploid regions found in the contigs 1–2204. MTP4BH contained about 0.1 Gbp from the 576 clones selected to represent the tetraploid and octoploid regions found in the 646 contigs in the 8000 and 9000 series. Each unique region of the genome should be represented once since build 4 did distinguish regions by ploidy.

MTP4BH inserts are present in the large (27.6 kbp) T-DNA vector pCLD04541. MTP4BH was suitable for plant transformation and functional genomics. BACs are readily transferred to *Agrobacterium tumefaciens *by triparental matings or electroporation (not shown). Plant cells, roots and whole regenerants resistant to kanamycin and containing BAC DNA as judged by PCR and southern hybridization have been selected.

Two plates of MTP4BH clones were derived from MTP2 and therefore were used for BAC-end sequencing, EST integration. The 12 plates of the MTP4BH were used for HICF and have been sent for BAC end sequencing. Plates 11 and 12 contained MTP2 BAC end sequenced clones [31]. A thirteenth plate contains 386 clone picked redundantly from 6 octoploid contigs to contrast with the low redundancy of the MTP4BH plates in uses. The plate has been used to test other methods of HICF fingerprinting (with Dr. MAC Luo, unpublished) and some clone pairs were used for DNA sequencing (With Dr. Gane Wu, unpublished) to determine what methods might separate highly conserved homeologous regions.

### MTP4E

MTP4E was 4,608 BAC clones with large inserts (mean 175 kbp) in the small (7.5 kbp) pECBAC1 vector. MTP4E was suitable for DNA sequencing. MTP4BH and MTP4E clones each encompassed about 0.8 Gbp, the 0.7 Gbp diploid regions and 0.05 Gbp each from the tetraploid and octoploid regions.

MTP4E was 12 plates that contained 4,608 *Eco*RI insert clones. The inserts of selected clones were of mean size 175 kbp as judged by 43.5 useful bands per fingerprint, and by agarose gel electrophoresis after digestion with *Not*I, or *Eco*RI. The 175 Kbp insert size is 18 Kbp larger than the mean insert sizes of the 2 contributing libraries suggesting large clones were selected. Therefore, MTP4E encompassed slightly more than 0.8 Gbp compared to the soybean haploid genome of 1 Gbp. MTP4E encompassed about 0.7 Gbp from the 4,032 clones selected from diploid regions found in the contigs 1–2204. MTP4E contained about 0.1 Gbp from the 576 clones selected to represent the unique portion of the 0.35 Gbp tetraploid and octoploid regions found in the 646 contigs in the 8000 and 9000 series. Each unique region of the genome should be represented once, since build 4 did distinguish regions by ploidy. A thirteenth plate contained 386 clones that were picked redundantly from 6 octoploid contigs to contrast with the low redundancy of the MTP4E plates in uses. MTP4E will be used for genome sequence by pooled genomic clone index.

#### Sources of error in build 2-based MTP construction

The high number of contaminated clones present during the build 2 automated contig procedure, and build 3 contig merges and splits created a high number (1040) of "Q" scores (Table 1). Each of these Q scores represents a clone in a contig that has bands that do not correctly match other clones in the contig. A macro-based procedure for contaminated and chimaeric clone identification was created [31]. From the list generated, each contig was inspected in order to find an alternate route across the contig without using these contaminated clones. The re-analysis of almost every contig created an MTP with an estimated 3–4 thousand extra clones. The inclusion of almost every chimaeric clone in every contig was balanced by the inclusion of a non-chimaeric BAC from the same region.

#### Benefits of MTP2

As the first available MTP for any legume, MTP2 clones were widely used. The *Hind*III and *Bam*HI-based plates were used for BAC end sequencing [31] and for high throughput EST array hybridization [23,32]. The primary benefits of this MTP were early availability and comprehensive nature through the 2 fold redundancy and use of all three libraries.

#### Sources of error in MTP4

Merging errors may have occurred when contig to contig merges were performed during build 4. Errors accumulate when aligning the contigs as dictated by the FPC program and the manual overlap of one contig with another. The FPC program tells the user how many clones match on each side of the contigs. For build 4 cutoff values of 1e-18 up to 1e-35 were used for merges, but the stringency of cutoff values depended on band number in the contig end clones. Since FPC does not give a merge distance (how much overlap should occur) a fixed overlap minimum of 25 bands was used for all merges (about 60% of the bands in the contig end clones) to promote consistent merging.

Acceptance of 3–4 band loss during MTP semi-automated selection allowed MTP4 to be as small, minimal and non-redundant as the fingerprint database could allow. MTP4 was 12 plates compared to the 38 plates of MTP2. The 3–4 bands lost per clone and ~300 clones removed, represents ~900 unique bands, or ~3.6 Mbp (<0.4%) of the genome that is not present in MTP4. However, small numbers of unique bands found in only one clone are often derived from chimaeric inserts composed of a small insert and a large insert from different genomic regions fused during ligation. Small insert contaminants are found in BAC libraries [1]. Therefore, the actual loss in genome coverage is expected to be significantly less than 3.6 Mbp

Separation of MTP4 into two parts MTP4BH and MTP4E reduced the representation of the MTP4 sets. The cloning efficiency of different soybean genomic regions varies. The restriction enzyme used [2] and the cloning vector selected [4,8] are major determinant in cloning efficiency. When both MTP4BH and MTP4E were used, the MTP4 genome representation exceeded MTP2 genome representation, but in 24 plates compared to 38 plates.

#### Benefits of build 4-based MTP

Separation of MTP4 into MTP4BH and MTP4E provided several benefits. Most contigs (98%) are represented in both sets, producing a robust MTP4. Antibiotic differences between the two vectors are more easily managed. Separation within MTP4BH included the previously sequenced *Hin*dIII and *Bam*HI clones that were placed in plates 11 and 12. Inclusion of the anchored clones within the un-sequenced clones would have resulted in over 1500 redundant end sequences. All plates except 11 and 12 were end sequenced (Genbank # DX406713 to DX414412; 7,700 sequences). If the 4,608 clones were sequenced, by STC, PGI or related methods [33-35] reads should be from non-redundant BACs that provide largely original sequence data.

The two additional plates of clones from several repeated contigs were included regardless of sequence status or inclusion MTP2. The clone sets provide a contrast to the low redundancy of the MTPs. Singleton clones are represented by the inclusion of 117 H and B BACs with band numbers in the range of 40–42. If non-chimaeric and unique, the clones represent ~4.6 million base pairs (Mbp) of DNA, or ~0.5% of available genomic DNA.

## Conclusion

**Figure 4:**
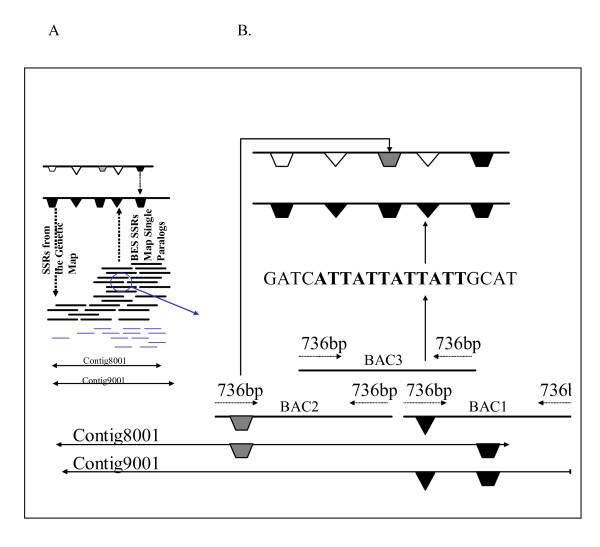
Diagram of the process to use repeat motifs in BAC end sequence to simultaneously anchor contigs and improve the genetic map. PanelA: Two contigs from a conserved duplicated region each contain the same mapped genetic markers (black symbols), 98% similar sequences (grey symbols) or distinct sequences (white). Panel B: New markers made from BES can distinguish the regions when amplifying genomic DNA [21] more efficiently than pooled BAC DNAs of reducing complexity among the anchors described previously [11].

The utility of minimum tiling paths has been demonstrated first by the use of subsets of the build 2-based MTP for BAC-end sequencing [31], EST integration [23,32], new micro-satellite integration into the physical map [21] and high information content fingerprinting (unpublished). In progress with the MTPs are further BAC end sequencing (for MTP4E), integration of fingerprints with an emerging physical map of soybean cv. Williams, development of new large DNA marker sets and genome sequence anchoring. The quality of the physical map will be tested by the release of the whole genome shotgun sequence by DOE in 2007–2008. Currently, BAC end derived microsatellite markers are providing excellent tools for placing contigs on the genetic and physical map and testing contigs already placed by satellite to BAC anchoring (Figure 4).

An interesting by-product of two, semi-overlapping MTPs is that a method for determining the efficiency and accuracy of a procedure can be provided, without the need to pre-select a set of clones. An example was provided by comparisons of MTP4 clones in which, by definition, most clones do not overlap, in a HICF fingerprint map. The inclusion of the same or an overlapping clone will provide answers in that procedure relating to accuracy, and repeatability. The use of an MTP based on a contig build also creates an efficient, well-spaced set of STS and markers [21]. The STS provide an opportunity to sample regions of the genome more equally than with markers located by genetic recombination alone.

Tools for MTP selection from paleopolyploid genomes will be developed further. The goal would be to completely automate the procedure for MTP selection using available FPC program output. A second goal was the creation of local automation protocols that would use available robotics to assemble new MTPs from future builds. The usefulness of creating a new MTP for each major build is demonstrated. New MTP development becomes exceedingly complex if previous MTP clones are used. The cost of duplication of effort can be high and should be avoided.

MTP2 and MTP4 are largely unordered. In future clones will be re-racked in map order so that neighboring clones in plate rows are neighbors on the soybean chromosomes. The chromosome arrays are useful for DNA sequencing by pooled genomic array [33] the analysis of synteny (30,35), to identify chromosomal rearrangements and to look at the timing of replication of chromosomes [20]. Among soybean germplasm, and other inbreeding legumes, chromosomal rearrangements are common.

Plant transformation with MTP4BH clones is an important future goal. In collaboration with Dr. Zhanjuan Zhang (U. of Missouri) the challenges that exist will be addressed. First, BIBAC vectors use the *npt*II gene for selection of kanamycin resistance. The *npt*II gene has allowed selection of soybean transformants and regenerants [36] but at lower efficiency than hygromycin or Roundup selectable markers. Second, the insert stability can depend on size, gene content, repeat content, ploidy and *Agrobacterium *genotype. In future functional complementation with dominant genes in Forrest will be attempted, including pubescence color [37] and significant soybean disease resistance and susceptibility alleles on isolated BIBAC clones [12,24].

## Materials and methods

BAC library master plates were obtained from the Texas A&M BAC repository in 1998 (B and H libraries) and 2001 (E library). Libraries were maintained at -80 C. Working copies of libraries were made in 384 well plates every 6 months. MTP libraries are maintained at SIUC and are available on request from Dr. D. A. Lightfoot.

### Build 2 minimum tiling path

**Figure 1:**
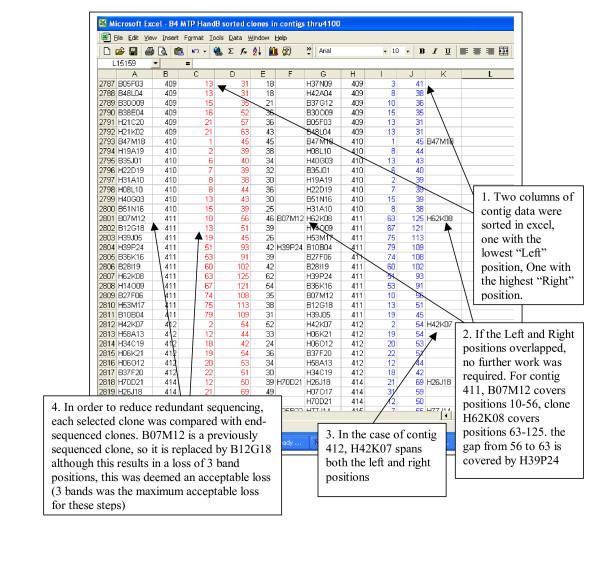
Example of procedure used to select clones for the build 4 minimum tiling path. Four columns are repeated twice. Each set contains the clone name (A & G columns), contig number (B & H columns), left position (C & I columns) and right position (D & J columns). The left group is sorted by contig, and leftmost position, while the right group is sorted by contig and rightmost position. Column E contains the number of bands for each clone in column A. Columns F and K contain clones selected for minimum tiling path construction.

Selection of clones was based on visual inspection of contigs. During this inspection, clear evidence of well contamination [5] was discovered (Figure 1; step 4; multiple *Bam*HI plate 17 clones). Each contig was processed to produce a list of clones. The clone lists were used to locate any BAC fingerprints that may have represented more than one clone or fragment of DNA. Each contig that had a potentially contaminated or chimaeric clone was re-examined, with a list being generated for all clones that were possibly contaminated. MTP generation is biased toward larger clones so that many potentially contaminated clones were discovered. Possible contaminants were retained in the dataset but a secondary route of clones covering the same region was added to the list.

The edited list of MTP2 clones was sorted alphabetically, in order to allow sequential picking from stock library plates. Groups of 96 clones were printed on an 8 × 12 columnar page. Pages were used as guides to pick from the 384 well plates into 96-well plates. As each plate was picked, it was incubated at 37°C, 230 rpm for 16–24 hours. Once the cell growth was clearly established, a 96 pin replicating tool was used to replicate the cells into the final 384 well master plate containing freeze media and LB broth. The 384 well plate was grown overnight at 37°C, with no shaking. After incubation, each plate was replicated, to create a working copy and stored at -80°C.

### Build 4 minimum tiling path

Selection of clones was based on numeric position generated by the FPC file program and represented in the FPC file as "Bands Left" and "Bands Right" [7]. The separate MTP4BH and MTP4E plates represented the best path within each contig using only clones from the selected libraries. Separation of MTP4 into two parts was driven by the different antibiotic resistances among the libraries and different uses of the plasmid vectors.

The MTP4BH and MTP4E lists of clones were sorted alphabetically, in order to allow sequential picking from stock library plates. Groups of 192 clones were printed on a 16 × 12 columnar page and used as a guide to pick into a 384-well plate (2 pages per plate). As each plate was inoculated, it was incubated overnight at 37°C, with no shaking. After incubation, each plate was replicated to create a working copy and stored at -80°C.

### Pulsed field gel electrophoresis

Isolation of BAC DNAs and restriction digestion with *Not*I, *Bam*HI, *Hind*III or *Eco*RI were as described in Meksem et al., [1]. Band sizes were estimated by comparison to lambda genome concatemers.

## Abbreviations

BAC, bacterial artificial chromosome (or large insert) clone.

BIBAC, T-DNA binary bacterial artificial chromosome (or large insert) clone.

Contig, contiguous overlapping set of BAC clones.

FPC, Finger Print Contig.

HICF, high information content fingerprinting

MTP, minimum tiling path.

## Competing interests

The author(s) declare that they have no competing interests.

## Authors' contributions

JS carried out the build development, participated in the MTP picking and drafted the manuscript. SY carried out the PFGE. CY, SK and JA participated in the anchoring and MTP picking. DAL conceived of the study, and participated in its design and coordination. All authors read and approved the final manuscript.
